# Microstructure, Wettability, Corrosion Resistance and Antibacterial Property of Cu-MTa_2_O_5_ Multilayer Composite Coatings with Different Cu Incorporation Contents

**DOI:** 10.3390/biom10010068

**Published:** 2019-12-31

**Authors:** Zeliang Ding, Yi Wang, Quan Zhou, Ziyu Ding, Jun Liu, Quanguo He, Haibo Zhang

**Affiliations:** 1 School of Mechanical Engineering, Hunan University of Technology, Zhuzhou 412007, China; wy15292222379@163.com (Y.W.); zhouquan321@163.com (Q.Z.); zhbtywzhb@163.com (H.Z.); 2 School of Packaging and Materials Engineering, Hunan University of Technology, Zhuzhou 412007, China; dingziyu0320@163.com; 3 School of Life Sciences and Chemistry, Hunan University of Technology, Zhuzhou 412007, China; liu.jun.1015@163.com

**Keywords:** implant material, corrosion resistance, antibacterial property, tantalum pentoxide, copper

## Abstract

Bacterial infection and toxic metal ions releasing are the challenges in the clinical application of Ti6Al4V alloy implant materials. Copper is a kind of long-acting, broad-spectrum and safe antibacterial element, and Ta_2_O_5_ has good corrosion resistance, wear-resistance and biocompatibility, they are considered and chosen as a potential coating candidate for implant surface modification. In this paper, magnetron sputtering technology was used to prepare copper doped Ta_2_O_5_ multilayer composite coating Cu-Ta_2_O_5_/Ta_2_O_5_/Ta_2_O_5_-TiO_2_/TiO_2_/Ti (Cu-MTa_2_O_5_ for short) on Ti6Al4V alloy surface, for studying the effect of copper incorporation on the microstructure, wettability, anticorrosion and antibacterial activities of the composite coating. The results showed that Cu-MTa_2_O_5_ coating obviously improves the hydrophobicity, corrosion resistance and antibacterial property of Ti6Al4V alloy. In the coating, both copper and Ta_2_O_5_ exhibit an amorphous structure and copper mainly presents as an oxidation state (Cu_2_O and CuO). With the increase of the doping amount of copper, the grain size, roughness, and hydrophobicity of the modified surface of Ti6Al4V alloy are increased. Electrochemical experiment results demonstrated that the corrosion resistance of Cu-MTa_2_O_5_ coated Ti6Al4V alloy slightly decreased with the increase of copper concentration, but this coating still acts strong anticorrosion protection for Ti6Al4V alloy. Moreover, the Cu-MTa_2_O_5_ coating can kill more than 97% of *Staphylococcus aureus* in 24 h, and the antibacterial rate increases with the increase of copper content. Therefore, Cu-MTa_2_O_5_ composite coating is a good candidate for improving anticorrosion and antibacterial properties of Ti6Al4V alloy implant medical devices.

## 1. Introduction

Reducing postoperative pain and the probability of postoperative complications, and speeding up the recovery of patients are both urgent problems to be addressed in the clinical application of implant materials [1]. Therefore, many scholars have carried out a lot of studies on improving the properties of implant materials such as stainless steel, titanium and its alloy, etc. [2,3,4]. Unfortunately, many problems, such as bacterial infection during surgery and toxic ion release during service, have not been effectively solved [5,6]. Since the corrosion and infection of implant materials are highly related to their surface properties, surface modification is considered to be the most effective method to overcome the problems in the clinical application of implant materials [7]. Among the availability of various surface modifications, the coating technique is often adopted, since it is not only a simple process, low-cost and easy industrialization way, but also enables the performance customization [1,8].

In recent years, various metal oxides such as titanium oxide, zirconia, alumina, silicon oxide, niobium oxide, and tantalum oxide have been used for surface modification coating of biomedical Ti6Al4V alloy, and their biological properties in vitro have also been investigated [4,9,10,11,12,13]. Among these oxides, tantalum oxide (Ta_2_O_5_) coating has recently drawn extensive attention due to its advantages such as excellent corrosion resistance and good biocompatibility and wear resistance [14,15]. At present, the main preparation methods of Ta_2_O_5_ coating include magnetron sputtering [16], sol-gel [17] and electron beam evaporation [18]. Ceramic coating with high purity, compact structure, uniform particle size, and good bonding performance, deposited by radio frequency sputtering ceramic target, has been widely used in aerospace, machinery, electronics, medical and other industries [19,20,21,22,23]. In addition, Ta_2_O_5_ coating also has antibacterial activity. For example, the bactericidal rate of Ta_2_O_5_ coating is 12% for *Escherichia coli* (*E. coli*) [24] and 30% for *Staphylococcus aureus* (*S. aureus*) [7]. However, the antibacterial effect of Ta_2_O_5_ coating is far from reaching the requirement of clinical application.

With the excellent bactericidal ability, copper shows strongly antibacterial to five kinds of bacteria such as *S. aureus*. Additionally, the bactericidal rate of copper towards a variety of bacteria can reach more than 90% or even 100%, and it presents a long-term, broad-spectrum and safety during this bactericidal process [25,26,27]. In 2008, copper and copper-bearing alloy were registered as the first effective metallic antibacterial material by the U.S. Environmental Protection Agency, which were considered to have the effect of killing 99.9% of bacteria within 2 h [28]. Moreover, copper, as an indispensable trace element in the human body, plays a vital role in maintaining the normal physiological ability of the body [29], such as the formation of osteoblasts in bone metabolism [30], regulating microvascular development, and accelerating skin wound healing [31,32]. Missing copper ions in the body may result in impaired bone growth and bone strength in animals [33]. It turned out that the copper, once added into TiO_2_, ZrO_2_ and other ceramic coatings can significantly improve the antibacterial properties of the coatings [34,35]. However, there are few reports on copper incorporation into Ta_2_O_5_ coating.

Meanwhile, for the good corrosion resistance, mechanical properties, and biocompatibility, Ti6Al4V titanium alloy is a research focus of implant materials in the fields of dentistry and orthopedics [15,36,37]. However, it was found by clinical studies that Ti6Al4V titanium alloy could be corroded by body fluids, releasing metal ions with the toxic and side effects, such as aluminum and vanadium, which could induce inflammation, allergy, poisoning and other reactions in human body, subsequently lead to the failure of implant surgery in serious cases [6,38]. Furthermore, because Ti6Al4V alloy itself has no fungicidal activity, during the surgery, bacteria could adhere to the surface of the implant to multiply to form biofilms, causing postoperative infection [39,40].

In our previous research [41], a Copper-incorporated Ta_2_O_5_ multilayer composite coating Cu-Ta_2_O_5_/Ta_2_O_5_/Ta_2_O_5_-TiO_2_/TiO_2_/Ti (Cu-MTa_2_O_5_ for short) on Ti6Al6V titanium alloy has been developed by magnetron sputtering technology. The microstructure, bonding strength, anticorrosion behavior and antibacterial activity of the coating were studied, revealing that the coating has excellent corrosion resistance and antibacterial performance, and bonding strength of 2.9 times that of monolayer Ta_2_O_5_ coating. For the further understanding of the influence of copper content on the microstructure and properties of the coating, the Cu-MTa_2_O_5_ multilayer composite coating with different copper content was prepared on Ti6Al4V by magnetron sputtering in this study. Scanning electron microscope (SEM), atomic force microscope (AFM), X-ray diffraction (XRD) and energy dispersive spectroscopy (EDS) and X-ray photoelectron spectroscopy (XPS) analysis were carried out for the microstructure characterization, phase composition and elemental chemical status of the coating. The wettability, anticorrosion and antibacterial properties of the coating were characterized by contact angle measurement instrument, electrochemical workstation and coating plate count method, respectively. As far as we know, this is the first study on the effect of copper doping on the microstructure and properties of Ta_2_O_5_ multilayer composite coatings on Ti6Al4V titanium alloys.

## 2. Materials and Methods

### 2.1. Coating Deposition

The substrates are silicon wafer (10 mm × 10 mm × 2 mm) and Ti6Al4V titanium alloy (10 mm × 10 mm × 0.6 mm). The composition of Ti6Al4V titanium alloy is Al, 6.8 wt %; V, 4.5 wt %; Fe, 0.3 wt %; O, 0.2 wt %; C, 0.1 wt %; N, 0.05 wt %; H, 0.015 wt %; and the surplus, Ti. Ta_2_O_5_, Ti and Cu target material (ZNNM., Beijing, China) have a size of Ø 75 mm × 4 mm and a purity of 99.99%. Argon is used as the working gas, oxygen as the reaction gas, their purity is 99.99%.

**Figure 1:**
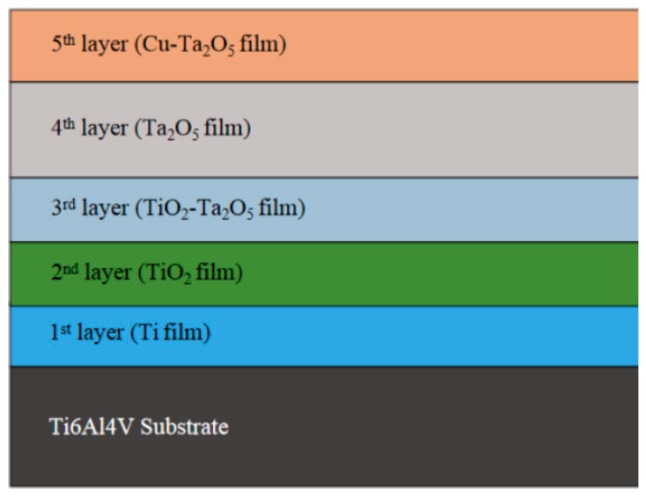
Scheme of Cu-MTa_2_O_5_ multilayer composite coatings.

**Figure 2:**
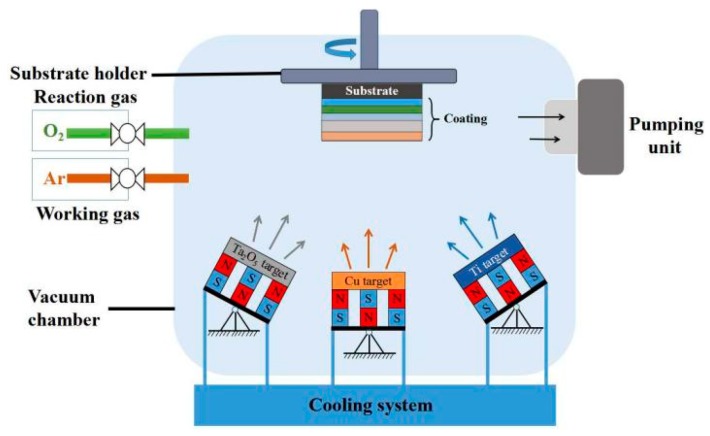
Schematic diagram of the magnetron sputtering system.

The structural diagram of Cu-MTa_2_O_5_ multilayer composite coating is presented in Figure 1. The first to the third layer of the composite coating is the intermediate transition layer, which is used to enhance the adhesion strength between the Ta_2_O_5_ coating and the Ti6Al4V substrate. The fourth layer, Ta_2_O_5_, and the fifth layer, Cu-Ta_2_O_5_, both are functional layers, which have functions of corrosion resistance and antibacterial effect respectively. Before depositing the coating, the Ti6Al4V substrate was successively ground with 240 to 2000 mesh SiC sandpaper, then polished for 10 min with 5 microns diamond paste and 500 nm alumina solutions respectively. Subsequently, the samples were washed under ultra-sonication for 15 min by acetone and anhydrous ethanol, respectively. After being dried by vacuum dryer, the samples were loaded into magnetron sputtering coating system (JCP-450, BJTN., Beijing, China) which can simultaneously install three targets and has three power sources of radio-frequency (RF), direct current (DC) and intermediate frequency (IF) (see Figure 2). The plasma was then employed to clean the substrates so as to remove the surface contamination and enhance surface activity.

**Figure 3:**
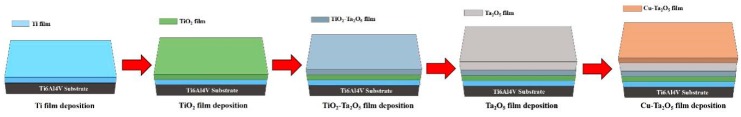
Schematic diagram of the deposition sequence of each film layer in Cu-MTa_2_O_5_ multilayer composite coatings.

Figure 3 shows that the deposition sequence of each film layer in Cu-MTa_2_O_5_ multilayer coating is Ti layer, TiO_2_ layer, TiO_2_-Ta_2_O_5_ layer, Ta_2_O_5_ layer and Cu-Ta_2_O_5_ layer in turn. Ti and Cu were deposited by direct current sputtering, TiO_2_ by direct current reactive sputtering, while Ta_2_O_5_ by radio frequency sputtering. Since the deposition rate of the metal film is positively proportional to sputtering power in an argon atmosphere, the doping amount of Cu in Cu-MTa_2_O_5_ composite coating can be adjusted by sputtering power of the Cu target. The sputtering power of Cu is set to be 0, 40, 60 and 80 W, and the corresponding codes of Cu-MTa_2_O_5_ multilayer composite coating samples are C0, C1, C2 and C3 respectively. The preparation parameters of the coating are shown in Table 1. The expected thickness of Ti film, TiO_2_ film, TiO_2_-Ta_2_O_5_ film, Ta_2_O_5_ film and Cu-Ta_2_O_5_ film are estimated to be about 200 nm, 50 nm, 100nm, 1000 nm and 250–450 nm, respectively. Silicon substrate coating samples were applied for characterization of coating surface and section, while Ti6Al4V substrate coating samples were used for performance study.

### 2.2. Coatings Characterization

Scanning electron microscope (Helios Nanolab G3 UC, Thermo Fisher Scientific Inc., USA) was employed to analyze the surface and interface micromorphology of coating specimens. The roughness of the coating surface was detected by AFM (EasyScan2, Switzerland). XRD (Ultima IV, Rigaku Corporation, Japan) was used to analyze the phase composition of the coating. The content of coating elements on the surface and cross-section of specimens was analyzed with EDS (Team Octane Plus, Ametek Group, USA). Element composition and chemical state of the coating surface were studied by XPS (EscaLab 250Xi, Thermo Fisher Scientific Inc., USA).

### 2.3. Contact Angle Tests

Generally, the contact angle (CA) is applied to assess the wettability of the sample surface [42]. At room temperature of 20 °C and ambient humidity of 50%, the CA measuring instrument (JC20001, POWEREACH, Shanghai, China) was used to determine the water contact angle of the sample. During the test, the liquid drops were placed on the sample surface with a standard microinjector and captured by a camera. In order to obtain accurate CA value, five different positions on the surface of the sample were measured, and the average value was taken as the test result.

### 2.4. Electrochemical Experiments

The corrosion properties of the sample were tested by PS-268A electrochemical detection system (SP-15/20A, Bio-Logic Science Instruments, France). During the test, simulated body fluid (SBF) with pH of 7.4 was used as electrolyte [43]. Platinum plate, saturated Ag/AgCl and target sample were used as counter electrode (CE), reference electrode (RE) and the working electrode (WE), respectively, where 1 cm^2^ of specimen surface area was exposed to SBF solution. The measurement range of the potentiodynamic polarization curve was −0.3~2.0 V, and the scanning rate was 1mV/s. Corrosion parameters including corrosion potential (*E_corr_*) and corrosion current (*I_corr_*) can be calculated from the potentiodynamic polarization curve by Tafel extrapolation. The polarization resistance (*R_p_*) can be calculated by the following formula [44,45]:

(1) $$R_{p}=\frac{\beta_{a}\times \beta_{c}}{2.3\times i_{corr}(\beta_{a}+\beta_{c})}$$

where, *β_a_* and *β_c_* are the Tafel slopes of the anode and cathode, respectively. All experiments were repeated three times and the experimental data were averaged.

### 2.5. Antibacterial Test

The plate counting method is the most common method for the quantitative evaluation of antibacterial properties of materials [41]. *S. aureus* is one of the common pathogens causing implant-related infection and implant inflammation [41]. In this study, the antibacterial effect of the sample on *S. aureus* (ATCC6538, Guangzhou Institute of Microbiology, Guangzhou, China) was tested by the plate counting method. Before the experiment, all specimens were sterilized by a vertical pressure steam sterilizer (parameters: 121 °C, 0.1 MPa, 30 min). The concentration of bacterial suspension was adjusted to 10^7^ CFU/mL by 0.9% of NaCl solution. 4 mL of bacterial suspension was injected into a sterile glass tube, the sample was placed in it and sealed, and then incubated in a shaking incubator for 24 h (ambient temperature was 37 °C). After that, the sample was taken out from the glass tube and the liquid was shaken for a uniform solution, then 100 μL of this bacterial solution was evenly coated on the agar plate and cultured in shaking incubator at 37 °C for 24 h. The automatic colony imaging analysis system (Sphere Flash, Barcelona, Spain) was used to take photos of the plate and count the active bacteria. The sterilizing rate (*X*) of the sample is calculated using the following formula [25]:

(2) $$X=\frac{M-N}{N}\times 100\%$$

where *M* and *N* is the average number of alive *S. aureus* colonies found on Ti6Al4V alloy and coating specimens, respectively.

## 3. Results and Discussion

### 3.1. Microstructure Characterization

**Figure 4:**
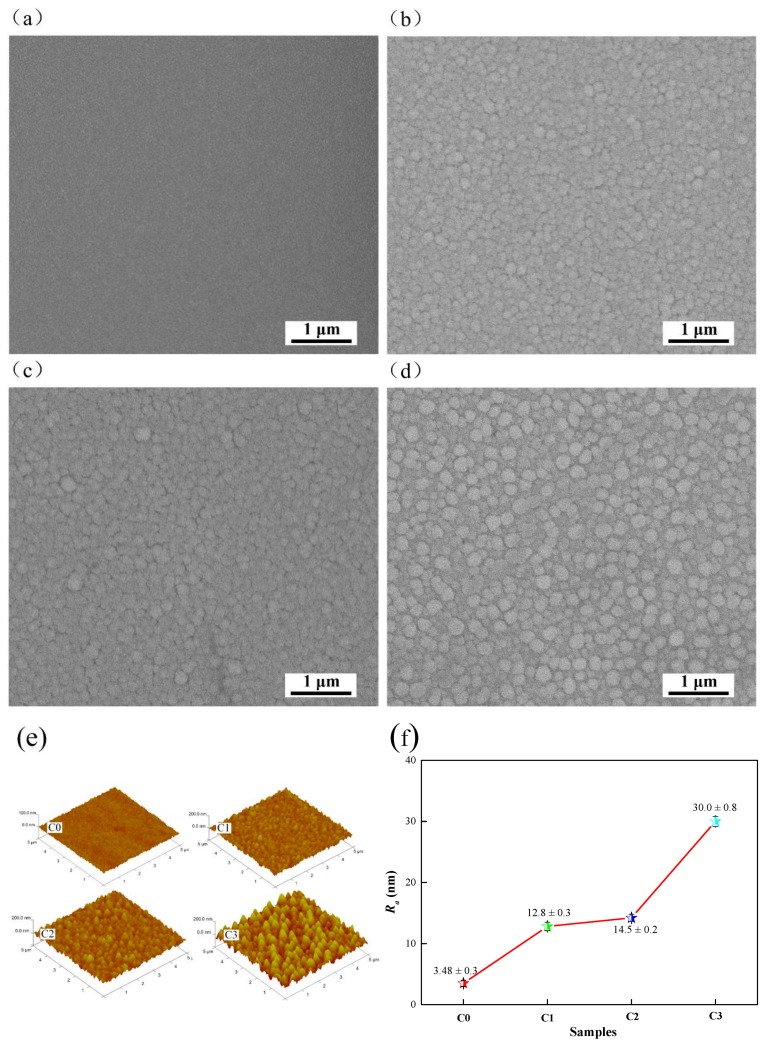
Scanning electron microscope (SEM) images: (**a**) C0, (**b**) C1, (**c**) C2 and (**d**) C3. (**e**) Atomic force microscope (AFM) images and (**f**) surface roughness.

Figure 4 displays SEM and AFM images of the surface of the coating samples. Figure 2a shows that Ta_2_O_5_ coating on the surface of the C0 sample is smooth, with small grain size and no obvious defects such as pores and cracks. With the increase of Cu incorporation into Ta_2_O_5_ coating, the grain diameter and grain boundary gap increased, and the microstructure density decreased (see Figure 4b–d). The increase of grain size is related to Cu grain agglomeration around Ta_2_O_5_ grain [46]. Figure 4e,f shows the AFM images of the coating sample surfaces with a scanning range of 5 μm × 5 μm and their corresponding roughness values. As shown in this Figure, the surface of all samples is composed of peak-type particles, the particle size is increased with the increasing of doped Cu element. The average values of surface roughness (*R_a_*) of the C0 sample without Cu doping is 3.48 ± 0.3 nm. The surface roughness of the C1 sample that mixed with 7.14 at % Cu is increased to 12.8 ± 0.3 nm. With the increase of Cu element incorporation, the surface roughness of C2 and C3 samples increased to 14.5 ± 0.2 nm and 30.0 ± 0.3 nm, respectively. Fewer doped elements in the coating can increase the compactness of the layer and reduce the surface roughness, while higher incorporated elements can increase the surface roughness [47]. These results show that the increase of Cu content will significantly affect the surface structure of the composite coating, inducing in the increase of grain size and roughness on the surface of these coatings.

**Figure 5:**
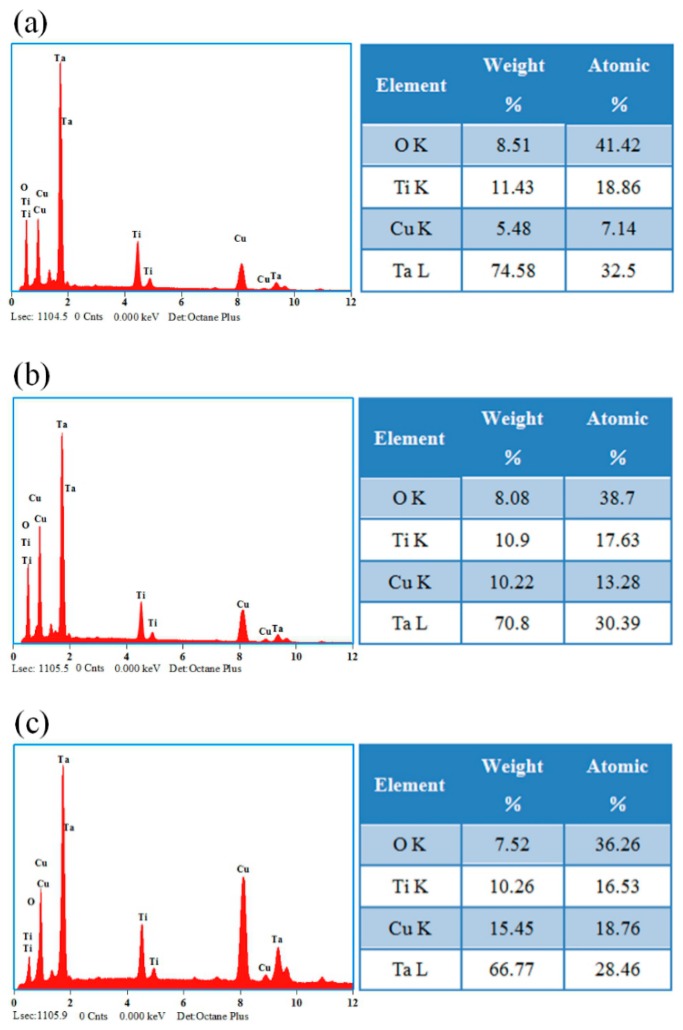
Energy dispersive spectroscopy (EDS) spectra images of coated samples: (**a**) C1, (**b**) C2 and (**c**) C3.

Figure 5 depicts the surface EDS analysis of coating samples. The outlayer of all three coating samples contains Cu, Ta and O elements. Among them, the incorporation of Cu in the C3 sample was the highest of 18.76 at %, followed by the C2 sample (13.28 at %), and the Cu content of the C1 sample was the lowest (7.14 at %). Because the coating deposited by magnetron sputtering technology, the deposition rate of the coating is positively proportional to the sputtering power of the target material [48]. Among these three samples, the C3 sample has the highest Cu target sputtering power (80 W), its Cu content is the highest.

**Figure 6:**
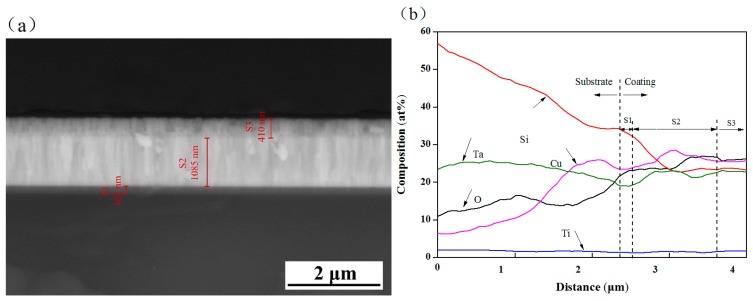
(**a**) Cross-section SEM image and (**b**) EDS line scan results along the thickness of C3 coating on the Si substrate.

Figure 6 shows the SEM image and EDS line scan results of cross-section of C3 coating sample. In Figure 6a, there are three obvious layers S1, S2 and S3 in the coating section. According to the coating structure (Figure 1) and the expected thickness of the individual film layer, it can be concluded that S1, S2 and S3 layers can be assigned to Ti film, TiO_2_/TiO_2_-Ta_2_O_5_/Ta_2_O_5_ film and Cu-Ta_2_O_5_ film, respectively. In the S2 region, there is no obvious interface among TiO_2_ film, TiO_2_-Ta_2_O_5_ film and Ta_2_O_5_ film, and no micropores or cracks appear. The two layers are basically integrated, which helps to improve the adhesion of adjacent layers. Figure 6b shows that Ta, Cu, Ti and O are contained in all S1, S2 and S3 of the coating. Among them, the rising trends of the contents of O, Ta and Cu in the coating are observed, while the Ti element was relatively stable. The fluctuation of element content is related to the region of the film and its components. These elements are distributed throughout the coating and diffused into the substrate, which helps to improve the chemical affinity between adjacent film layers and form the metallurgical bonding interface for reducing interfacial stress and improving coating bonding strength.

**Figure 7:**
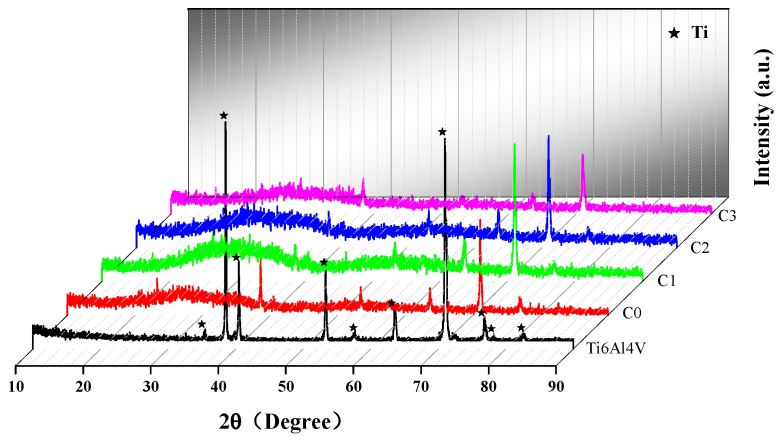
XRD patterns of coated and un-coatedTi6Al4V.

Figure 7 displays the XRD images of Ti6Al4V and coated Ti6Al4V samples. No characteristic peaks of Cu and Ta_2_O_5_ appear in the XRD patterns of all the coating samples, indicating that Cu and Ta_2_O_5_ in the coatings are amorphous structure [49]. The appearance of an amorphous structure could be related to low deposition temperature and low sputtering power [50,51]. Previous studies have found that when the annealing temperature is about 800 °C, the Ta_2_O_5_ film deposited by sputtering at room temperature starts to crystallize, while the crystallization temperature of Cu film is above 300 °C [52,53]. In addition, the diffraction peak of Ti appears in the XRD pattern, which may be due to the porous structure and small thickness of the coating, causing Ti to diffuse from the intermediate layer or substrate to the coating surface. With the increase of Cu incorporation, the coating thickness increases, and the strength of the Ti peak gradually decreases. The chemical valence of the elements in the coating needs to be further determined by XPS testing.

**Figure 8:**
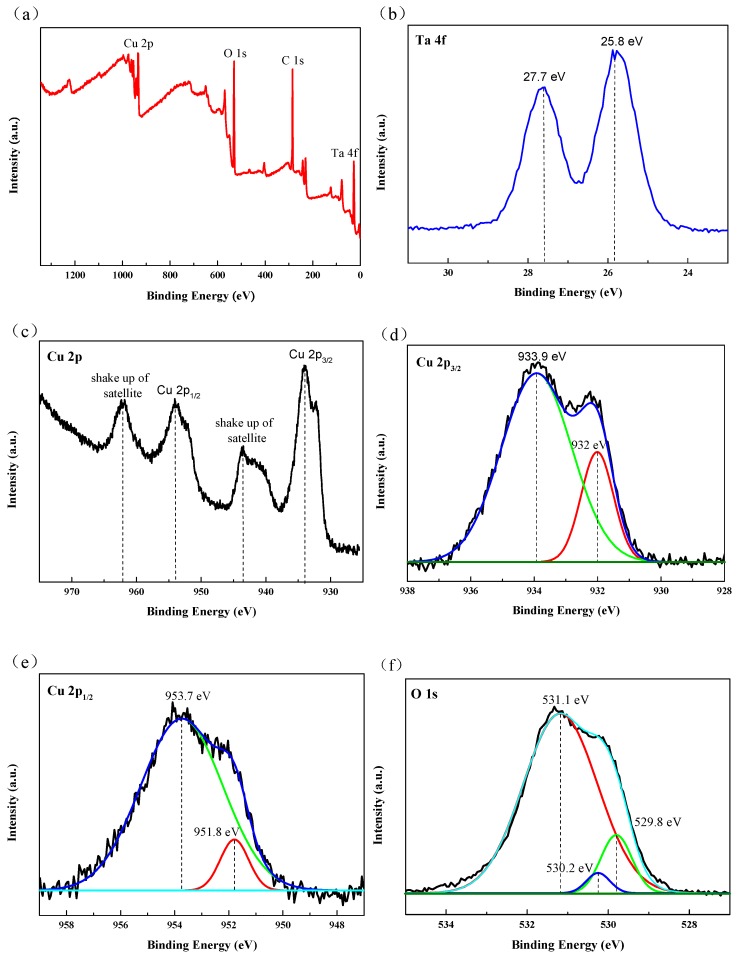
(**a**) XPS survey spectrum and (**b**) Ta 4f, (**c**–**e**) Cu 2p, (**f**) O 1s high-resolution spectra of the C3 coating sample.

Figure 8 displays the XPS spectra of the C3 sample. The full XPS spectrum in Figure 8a shows the peaks of Cu 2p, O 1s and Ta 4f, indicating the presence of Cu, O and Ta on the surface of the C3 sample. In the high-resolution spectrum of Ta 4f (Figure 8b), two peaks located at the binding energy position of 25.8 eV and 27.7 eV correspond to the characteristic peaks of Ta 4f_7/2_ and Ta 4f_5/2_, respectively, indicating that the chemical state of Ta on the surface of C3 sample is Ta_2_O_5_ [17].

Two characteristic peaks of Cu 2p_3/2_ and Cu 2p_1/2_ appear in the high-resolution spectrum of Cu 2p shown in Figure 8c, and are deconvolved to obtain the high-resolution spectrum of Figure 8d and Figure 8e, respectively. In Figure 8d, the peak at the binding energy of 933.9 eV is related to CuO, while the peak of 932 eV is related to Cu or Cu_2_O [34]. The peak located at 951.8 eV could be ascribed to the Cu 2p_1/2_ from Cu or Cu_2_O in the high-resolution spectrum of Figure 8e, while the peak located at 953.7 eV is from CuO. In addition, two satellite peaks are also observed, which are attributed to CuO (Figure 8c), which further confirms the existence of CuO in the coating [33]. Since copper is easy to be oxidized, the oxidation state of copper is observed in this Cu doped in C3 coating.

In high-resolution XPS spectrum of O 1s (Figure 8f), three deconvolution peaks at binding energy position of 531.3 eV, 530.2 eV and 529.8 eV could be attributed to Ta_2_O_5_, CuO and Cu_2_O, respectively [54,55,56]. These results show that the chemical state of the Ta element in Cu-MTa_2_O_5_ composite coating is Ta_2_O_5_, while both CuO and Cu_2_O existed as state Cu elements. More importantly, Ta_2_O_5_ can improve the corrosion resistance and biocompatibility of the implant material [15], while CuO and Cu_2_O can improve the antibacterial activity of the implant material [34].

### 3.2. Wettability

**Figure 9:**
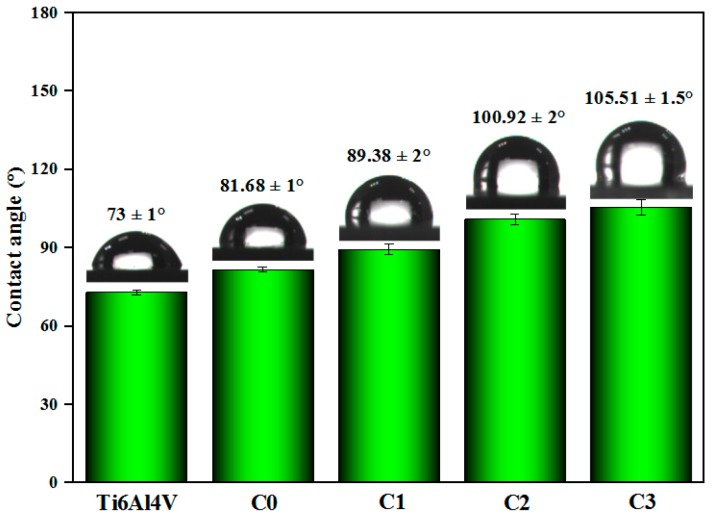
Contact angle value and water droplet photo of coated and uncoated samples.

Wettability is one of the important surface properties of implant materials that affect cell/bacterial response, which is generally evaluated by testing the surface contact angle [41]. Figure 9 is the test result of the water contact angle on the surface of the sample. The contact angles of coated samples are all greater than that of uncoated Ti6Al4V alloy (73 ± 1°). The contact angle of C0 sample without adding Cu was 81.68 ± 1°. With the increase of Cu incorporation in the coating, the contact angle is gradually increased. The contact angle of the C3 sample with the most Cu content was 105.51 ± 1.5°. This is because the roughness surface of this sample, it is increased with the adding of Cu element, and the contact angle is in direct proportion to the surface roughness [57].

### 3.3. Anticorrosion Property

**Figure 10:**
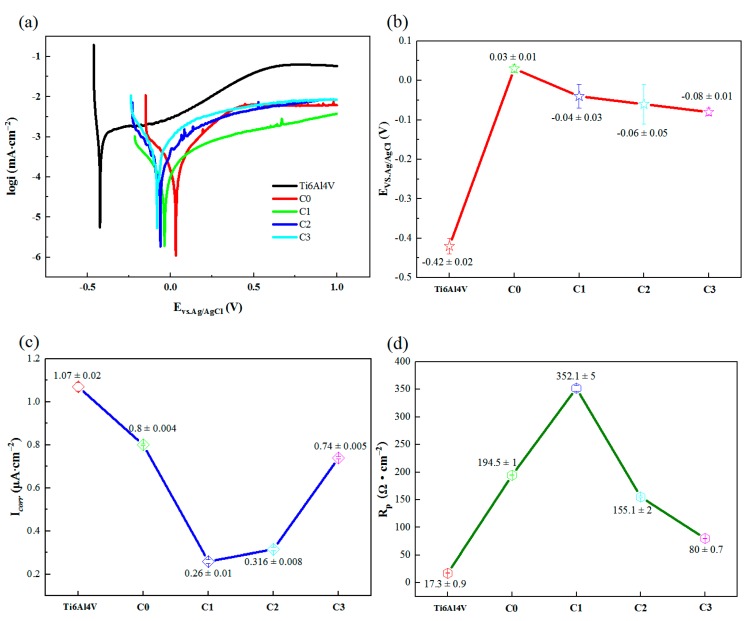
Potentiodynamic polarization curves (**a**) and corrosion parameters of coated and uncoated Ti6Al4V: (**b**) *E_corr_*, (**c**) *I_corr_*, (**d**) *R_p_*.

Figure 10a shows the electrochemical potentiodynamic polarization curve of the specimens in SBF, Figure 10b,c presents the corrosion parameters corresponding to Figure 10a obtained by Tafel extrapolation. The *E_corr_* value of bare Ti6Al4V is −0.42 V, while the *E_corr_* values of all coating specimens are higher than that of bare Ti6Al4V. However, the *E_corr_* values of coating specimens decrease with the increase of Cu content, where C0 (0.03 ± 0.01 V) has the highest *E_corr_* value, followed by C1 (−0.04 ± 0.03 V), C2 (−0.06 ± 0.05 V) and C3 (−0.08 ± 0.01 V).

In addition, the *I_corr_* value of Ti6Al4V alloy (1.07 ± 0.02 μA/cm^2^) was the highest among all specimens, while the *I_corr_* values of coating specimens decrease first and then increases with the increase of Cu incorporation, and the *I_corr_* value of C1 sample (0.26 ± 0.01 μA/cm^2^) was the lowest. In Figure 10d, Ti6Al4V alloy shows the smallest *R_p_* value, while the C1 sample with the least addition of Cu has the highest *R_p_* value. Higher *E_corr_*, smaller *I_corr_* and larger *R_p_* may make the materials more resistant to corrosion [15]. These results indicate that the Cu-MTa_2_O_5_ composite coating has a good effect of corrosion protection on Ti6Al4V alloy. The improvement of anticorrosion property for these coating specimens is attributed to the excellent chemical stability of Ta_2_O_5_ ceramic coating [58]. A small amount of Cu can improve the densification of the coating, and prevent the substrate from eroding by corrosion ions, and enhance the anticorrosion property of the substrate. However, since Cu can be easily oxidized, when the addition of Cu in the coating is relatively large, more copper ion is released into the corrosive solution, leading to a reduction in anticorrosion property of the sample [59].

### 3.4. Antibacterial Properties

**Figure 11:**
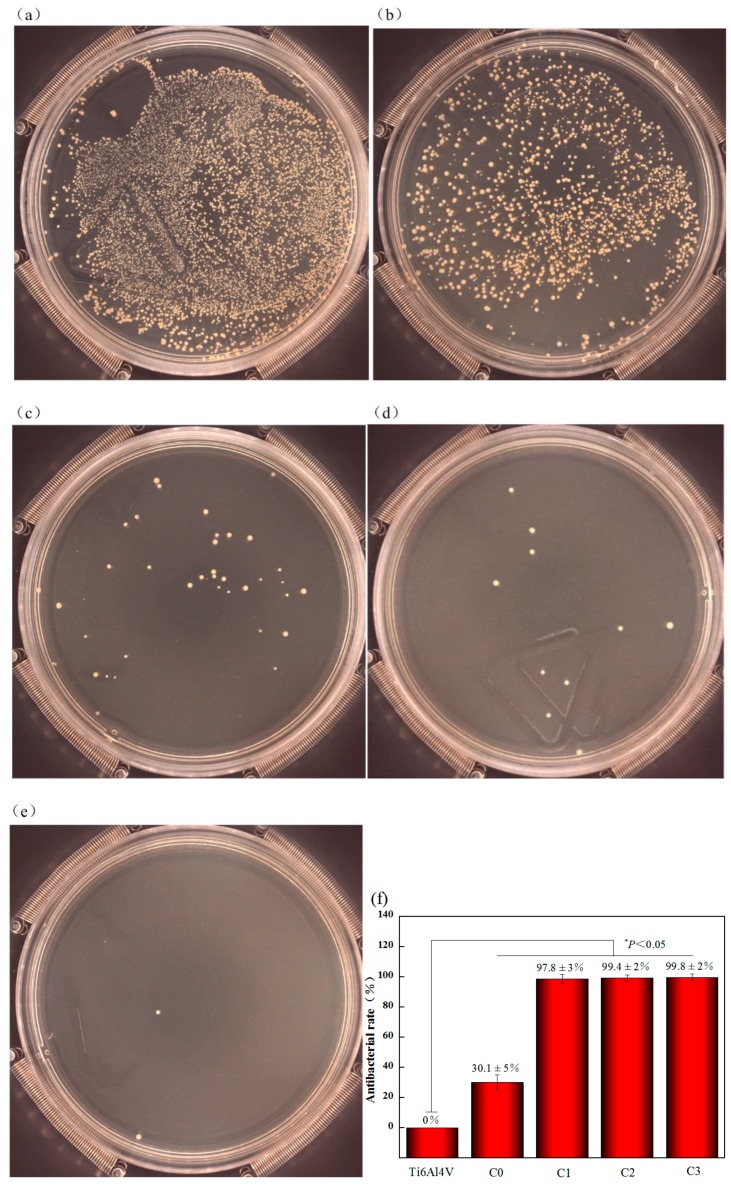
Live *S. aureus* after co-cultured with different samples at 37 °C for 24 h: (**a**) Ti6Al4V, (**b**) C0, (**c**) C1, (**d**) C2, (**e**) C3 and (**f**) Antibacterial rates of all samples toward *S. aureus*.

Figure 11 shows the appearance of *S. aureus* colony on the surface of the agar plate. The Figure shows the characteristics of bacterial colonies after the sample is co-cultured with bacterial liquid for 24 h and then cultured on the surface of the agar plate for 24 h. The largest number of bacterial colonies is observed in the polished Ti6Al4V sample (Figure 11a), the second one is C0 sample. But the bacterial colonies cultured with Cu doped samples C1, C2 and C3 are significantly reduced, with only 40, 10 and 2 of strains, respectively, and the antibacterial rate is more than 90% (Figure 11c,d). These results show that Ta_2_O_5_ coating has a certain bactericidal ability, while Cu doped Ta_2_O_5_ coating has excellent antibacterial properties by compared with pure polished Ti6Al4V alloy.

**Figure 12:**
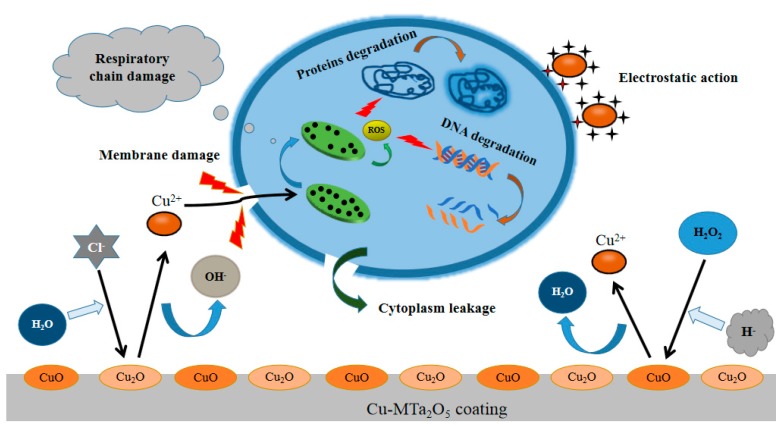
Schematic diagram of possible sterilization mechanism of Cu ions released by Cu-MTa_2_O_5_ coating.

The antibacterial effect of Cu-containing coating is attributed to Cu ions dissolved from the coating [34]. As shown in Figure 12, when the Cu-bearing coating sample is immersed in the bacterial solution, Cu ions are released from the coating surface and diffuse into the solution. These Cu ions are adsorbed on the cell membrane of bacteria through electrostatic action, which limits the activity of bacteria, inducing metabolic disorders and cell death [60]. Moreover, after contact with bacteria, Cu ions penetrate the membrane into the cell, which destroys membrane integrity and leads to cell death due to the leakage of the cytoplasm such as proteins and reducing sugars [61]. In addition, Cu ions entering the cell can damage the respiratory chain of the bacterial, cause the production of a large amount of ROS, degradation of DNA and proteins, and ultimately cell death [26]. The antibacterial activity of C0 sample is related to the amorphous structure [62] and the release of Ta^5+^ ions [24]. But at present, there is few researches focus on the antibacterial mechanism of Ta_2_O_5_, and its detailed mechanism needs to be further investigated carefully.

## 4. Conclusions

In conclusion, Cu-Ta_2_O_5_/Ta_2_O_5_/Ta_2_O_5_-TiO_2_/TiO_2_/Ti (Cu-MTa_2_O_5_ for short) multilayer composite coating with different Cu incorporation content is fabricated on Ti6Al4V alloy by magnetron sputtering. The effect of Cu content on microstructure, wettability, corrosion resistance and antibacterial activity of the composite coating was investigated. The surface of the Ta_2_O_5_ multilayer composite coating with or without copper had greater surface roughness and water contact angle than Ti6Al4V alloy. With the increase of copper content, the surface roughness and hydrophobicity of the copper-containing coating samples were increased. More importantly, although the corrosion potential of the copper-doped coating samples is slightly lower than that of copper-undoped coating sample, it shows smaller corrosion current and is able to effectively prevent the corrosion medium from attacking the Ti6Al4V alloy. In addition, the antibacterial rate of Cu-MTa_2_O_5_ multilayer composite coating containing 7.14 at% copper reached 97.8 ± 3%, and improved with the increase of copper content. Therefore, this study can provide useful help in the multi-functionalization of Ti6Al4V alloy surface modification for biomedical implant applications. However, further effort, such as preparation parameter optimization and biocompatibility assessment of Cu-MTa_2_O_5_ coatings, needs to be carried out.

## Figures and Tables

**Table 1:** Coating preparation parameters.

Layer Number	Coating Materials		Sputtering Power (W)	Deposition Time (min)	Gas Flow (sccm)	
					Ar	O_2_
1st layer	Ti		200	8	20	
2nd layer	TiO_2_		200	8	16	4
3rd layer	TiO_2_-Ta_2_O_5_	TiO_2_	200	8	20	5
		Ta_2_O_5_	200			
4th layer	Ta_2_O_5_		200	105	20	
5th layer	Cu-Ta_2_O_5_	Cu	0, 40, 60, 80	15	20	
		Ta_2_O_5_	200			
